# Improved gestational diabetes screening protocol

**DOI:** 10.1515/almed-2020-0072

**Published:** 2021-02-17

**Authors:** Miguel Calero Rojas, Aurora Jurado Roger, Marta Gutiérrez Grúa, Lourdes de la Peña Carretero, Victoria Romero Sotomayor, Javier López Braos, Federico Izquierdo Carrasco, Luis Herrero Tabanera, Carmen Moreno Aguilar

**Affiliations:** Obstetrics and Gynaecology Unit, Hospital Infanta Margarita, Cabra, Spain; Immunlogy and Allergy Unit, Hospital Universitario Reina Sofía-IMIBIC, Córdoba, Spain; Laboratory Unit, Hospital Infanta Margarita, Cabra, Spain; Operation Control Unit, Hospital Infanta Margarita, Cabra, Spain

**Keywords:** adverse events, gestational diabetes, glucose challenge test, glycated haemoglobin, screening

## Abstract

**Objectives:**

This work aimed to assess the diagnostic validity of two approaches for the screening of gestational diabetes mellitus (GDM) with less discomfort for pregnant women.

**Methods:**

A prospective diagnostic validation study was conducted with 2007 pregnant women. According to risk factors for GDM, women were classified into high-risk and low-risk groups. The current diagnostic procedure, based on oral glucose overload, was followed; simultaneously HbA_1c_ was tested and an algorithm combining both biomarkers was applied.

**Results:**

In the low-risk group, the Glucose challenge test (GCT) showed a higher area under the curve (AUC 0.953; 95% CI 0.915–0.992) than the HbA_1c_ test (0.688; 95% CI 0.541–0.834). The best GCT cut-off, 153.5 mg/dL (8.52 mmol/L), showed higher diagnostic validity than that for HbA_1c_, 28 mmol/mol (4.75%), and that the algorithm using both tests. In the high-risk group, the GCT showed better diagnostic performance than the HbA_1c_ and the algorithm; the optimal GCT cut-offs were higher than those recommended in current protocols. 13th week: GCT AUC 0.882 (95% CI 0.843–0.921), HbA_1c_ AUC 0.624 (95% CI 0.562–0.686), GCT cut-off 140.5 mg/dL (7.8 mmol/L), HbA_1c_ cut-off 33 mmol/mol (5.15%). 24th week: GCT AUC 0.944 (95% CI 0.925–0.962), HbA_1c_ AUC 0.642 (95% CI 0.575–0.709), GCT cut-off, 145.5 mg/dL (8.08 mmol/L), HbA_1c_ cut-off 29 mmol/mol (4.85%).

**Conclusions:**

The GDM diagnostic approach using as the first step the GCT with higher cut-offs showed the best diagnostic validity. Applying these thresholds, 55.6 and 13.7% of 100 g. Oral glucose overloads would have been avoided in low-risk and high-risk pregnant women.

## Introduction

Gestational diabetes mellitus (GDM) has been defined for years as any degree of glucose intolerance with onset or first recognition during pregnancy [1] and is considered an increasing health problem worldwide. More recently, this definition has been modified by the World Health Organization [2] and the American Diabetes Association [3], applying the term GDM for those cases diagnosed in the second or third trimester of pregnancy that is not Type 1 or Type 2 diabetes. However, these new criteria are not universally accepted and, controversy on the definition and the diagnostic workflow remains a matter of debate, [4], [5], [6], [7].

Per the classical definition of GDM, the prevalence rates range from 2–17% [7], [ 8]. In Spain, the likelihood of developing GDM is 8.8% [9].

The Spanish Group for Diabetes and Pregnancy (GEDE) classifies pregnant women into two groups: high-risk and moderate/low-risk. High-risk is considered when at least one of the following factors is present: age (>35 years old), obesity (BMI>30 kg/m^2^), history of GDM, first-degree relatives with diabetes mellitus and, history of macrosomia [10].

The validated approach for diagnosing GDM in Spain is a two-step strategy using the oral glucose challenge test (GCT) and the oral glucose tolerance test (OGTT). The GCT is performed at week 24 of gestation (week 13 if there is a risk factor) and if this test is positive (≥140 mg/dL; ≥7.77 mmol/L), an OGTT is performed. The GCT consists of the oral administration of 50 g of glucose and measurement 1 h later. The OGTT consists of the oral administration of 100 g glucose, which is measured at baseline levels and after 1, 2 and 3 h [10], [ 11]. These procedures are poorly tolerated by pregnant women, who often have vomiting that prevents the process from ending. Additionally, other drawbacks of these procedures are the lead time to complete the OGTT, frequent vomiting that compromises the technique and pre-analytical glycolysis in the plasma glucose samples that can underestimate the GDM diagnosis [4], [6], [12], [13], [14], [15], [16].

In 2009, the International Expert Committee on Diabetes recommended the glycated haemoglobin A (HbA_1c_) assay as the choice test for the chronic management of diabetes [16]. The HbA_1c_ test has several advantages over assays based on plasma glucose levels, such as standardization, better correlation with long-term adverse events, decreased biological variability, less pre-analytical errors, no need for timed sampling and less affection by acute perturbations in glucose levels. Nevertheless, for the diagnosis of diabetes during pregnancy, a period with changes in erythrocyte turnover, the diagnostic accuracy of HbA_1c_ could be affected and glucose measurement based tests are recommended [7], [ 17].

Subsequent articles have shown that the HbA_1c_ test could be a useful technique for diagnosing diabetes mellitus in high-risk individuals [18], [ 19]. However, few studies have validated the use of HbA_1c_ for diagnosing GDM [20], [21], [22], [23], [24] and its use remains controversial [25].

Therefore, the purpose of this study is to assess the diagnostic validity of two new approaches for the screening of GDM, with the least discomfort for pregnant women.

## Materials and methods

This prospective study was carried out in a tertiary hospital (Infanta Margarita Hospital). The study population comprised all pregnant women treated at the hospital and its reference area (southern Spain) for a period of three years. The research related to human use has been complied with all the relevant national regulations, institutional policies and in accordance the tenets of the Helsinki Declaration, was approved by the local institutional Ethics Committee of Reina Sofía Hospital (Córdoba) and was financially supported by a grant of The National Institute of Health Carlos III (ISCIII) (PI11 01064).

Assuming a prevalence of 10%, 85% sensitivity, 85% specificity and allowable percentage type II errors of 5% at a 95% significance level, a sample of 1970 eligible participants was calculated using Epidat 4.1 software (Conselleria de Sanidade Galega).

All pregnant women who attended the Obstetric Unit from September 2011 to September 2014 and accepted to participate in the study were included. Participants signed informed consent. Those women affected with pregestational diabetes, haemoglobinopathies or any condition with an increased red cell turnover (anaemia, transfusion) were excluded.

A total of 2,270 pregnant women were initially included. A total of 221 were excluded for several reasons including, tracking losses (134), miscarriage (39), tracking in a private institution (31) and pitfalls of demographic data (17). Moreover, 42 participants were excluded from the final analysis due to transfusions (35), pregestational diabetes (3), haemoglobinopathies (2) and leaving the study (2). A total of 2007 pregnant women were finally included in the statistical analysis.

Study participants underwent a physical examination and were given a structured questionnaire to identify risk factors for GDM: age >35 years, history of macrosomia, obesity (BMI>30 kg/m^2^), history of GDM and first-degree relatives with diabetes mellitus and ethnic origin with a high prevalence of diabetes mellitus (Black, Latino American or Asian women).

A 50 g GCT and HbA_1c_ test were performed between 24th and 26th weeks of pregnancy (GCT-24w; HbA_1c_-24w). If positive (≥140 mg/dL; ≥7.77 mmol/L), the 100 g OGTT was performed. An OGTT was considered positive if ≥105 mg/dL (≥5.83 mmol/L) (baseline), ≥190 mg/dL (≥10.55 mmol/L) (1 h), ≥165 mg/dL (≥9.16 mmol/L) (2 h), ≥145 mg/dL (≥8.05 mmol/L) (3 h) [6], [ 7]. GDM was diagnosed if any of the following criteria were met: fasting plasma glucose ≥126 mg/dL (≥6.99 mmol/L) (measured twice), random glycaemia ≥200 mg/dL (≥11.1 mg/L) and two or more altered points in the OGTT (per GEDE [10] and National Diabetes Data Group criteria [11]). Women with risk factors for GDM were evaluated twice with the same schedule: at week 13 of gestation (GCT-13w; HbA_1c_-13w) and between week 24 and 26.

Depending on their epidemiological history, clinical, and laboratory findings, the participants were allocated to different groups by obstetrical consultants, who were blinded to the HbA_1c_ results.

Different laboratory technicians and physicians oversaw carrying out the clinical analysis of the glucose test and the HbA_1c_ test and were blinded to the result of the alternative test and the women group inclusion.

**Laboratory procedures** (see Supplemental Material).
**Statistical analysis** (see Supplemental Material).
**Strategies for diagnosing GDM** (see Supplemental Material).

## Results

The frequency distribution of the continuous variables was non-Gaussian in all cohorts (the entire population, women with risk factors and women without risk factors). The data of the descriptive analysis are shown in Table 1.

**Table 1:** Distribution of clinical variables of the study population.

Clinical variables	All women (n=2,007)	Low-risk group (n=1,054)	High-risk group (n=953)
	Median (IR^a^)	Median (IR)	Median (IR)
Age, years	31 (28–35)	29 (26–32)	34 (31–37)
GCT-13w^b^, mg/dL	na^c^	na	109 (89–131)
GCT-13w, mmol/L	na	na	6.05 (4.94–7.27)
HbA_1c_-13w^d^, mmol/mol	na	na	31.1 (29–33.3)
HbA_1c_-13w, %	na	na	5 (4.8–5.2)
GCT-24w^e^, mg/dL	115 (96–138)	110 (93–132)	122 (102–144)
GCT-24w, mmol/L	6.38 (5.33–7.66)	6.11 (5.16–7.33)	6.77 (5.66–7.99)
HbA_1c_-24w^f^, mmol/mol	29 (25.7–31.1)	28 (25.7–31.1)	29 (26.8–32.2)
HbA_1c_-24w, %	4.8 (4.5–5)	4.7 (4.5–5)	4.8 (4.6–5.1)
	**%**	**%**	**%**
GDM^g^ prevalence	5.7	1.8	10
Risk factors	47.5	0	1
Age>35 years old	22.5	0	47.4
BMI^h^>30 kg/m^2^	13.7	0	28.8
Macrosomia	2.2	0	4.7
PH^i^ of GD	3.4	0	7.1
FH^j^ of diabetes	24.7	0	51.9
Ethnic origin at risk	0.1	0	0.2

^a^IR, interquartile rank; ^b^GCT-13w, 50 g glucose challenge test at week 13th; ^c^na, not applicable; ^d^HbA_1c_-13w, glycated haemoglobin test at week 13th; ^e^GCT-24w, 50 g glucose challenge test at week 24th; ^f^HbA_1c_-24w, glycated haemoglobin test at week 24th; ^g^GDM, gestational diabetes mellitus; ^h^BMI, body mass index; ^i^PH, personal history; ^j^FH, familiar history.

### Analysis of the whole population

The prevalence of GDM in the whole population was 5.7%. The medians of the variables age, GCT-24w and HbA_1c_-24w were significantly higher in women with risk factors (p<0.001). Pregnant women who developed GDM were significantly older and had higher levels in the GCT and HbA_1c_ test (p<0.001) at week 24. The presence of risk factors, in detail being over 35 years old, a BMI>30 kg/m^2^, previous GDM and a family history of diabetes mellitus, were significantly more common among pregnant women who developed GDM than in those who did not (p<0.001) (Tables 2 and 3). Although 79 women were born in another country (20 different nationalities), only two of them were non-Caucasian people.

**Table 2:** Comparison of the continuous variables between pregnant women who developed (cases) and who did not develop GDM^a^.

Population	Continuous variables	Cases: median (IR^b^)	Controls: median (IR)	p-Value
**All women**	Age, years	34 (30.75–37)	31 (28–34)	<0.001
	GCT-24w^c^, mg/dL	168 (156–187.5)	114 (96–135)	<0.001
	GCT-24w, mmol/L	9.32 (8.69–10.41)	6.33 (5.33–7.49)	
	HbA_1c_-24w^d^, mmol/mol	31(28–36)	29(26–31)	<0.001
	HbA_1c_-24w, %	5 (4.7–5.4)	4.8 (4.5–5)	
**Women without risk factors**	Age, years	30 (28–33)	29 (26–32)	ns^e^
	GCT-24w, mg/dL	167 (160–180)	110 (92–130)	<0.001
	GCT-24w, mmol/L	9.27 (8.88–9.99)	6.11 (5.11–7.22)	
	HbA_1c_-24w, mmol/mol	31 (29–37)	28 (26–31)	0.006
	HbA_1c_-24w, %	5 (4.77–5.5)	4.7 (4.5–5)	
**Women with risk factors**	Age, years	35 (31–37)	34 (31–37)	ns
	GCT-13w^f^, mg/dL	163 (134.5–184)	6106 (89–125)	<0.001
	GCT-13w, mmol/L	9.05 (7.46–10.21)	5.88 (4.77–6.94)	
	HbA_1c_-13w^g^, mmol/mol	33 (30–36)	31 (29–33)	<0.001
	HbA_1c_-13w, %	5.2(4.9–5.4)	5(4.8–5.2)	
	GCT-24w, mg/dL	168 (154.7–189.7)	119.5 (100–139)	<0.001
	GCT-24w, mmol/L	9.32 (6.59–10.53)	6.63 (5.55–7.71)	
	HbA_1c_-24w, mmol/mol	31 (28–34)	29 (27–32)	<0.001
	HbA_1c_-24w, %	5 (4.7–5.3)	4.8 (4.6–5.1)	

^a^GDM, gestational diabetes mellitus; ^b^IR, interquartile range; ^c^GCT-24w, 50 g glucose challenge test at week 24th; ^d^HbA_1c_-24w, glycated haemoglobin test at week 24th; ^e^ns, not significant; ^f^GCT-13w, 50 g glucose challenge test at week 13th; ^g^HbA_1c_-13w, glycated haemoglobin test at week 13th.

**Table 3:** Odds ratios for each risk factor (discrete variables) for pregnant women who developed (cases) and did not develop GDM^a^.

Population	Discrete variables	OR^b^	95% CI^c^	p-Value
All women	To have risk factors	6.03	3.65–9.95	<0.001
	Age >35 years	2.11	1.42–3.13	<0.001
	Macrosomia			ns^d^
	BMI^e^>30 kg/m^2^	3.83	2.54–5.76	<0.001
	PH^f^ of GDM	13.02	7.63–22.2	<0.001
	FH^g^ of GDM	2.44	1.66–3.59	<0.001
Women with risk factors	Age >35 years			ns
	Macrosomia			ns
	BMI >30 kg/m^2^	1.93	1.25–2.99	<0.001
	PH of GDM	7.32	4.23–12.65	<0.001
	FH of GDM			ns

^a^GDM, gestational diabetes mellitus; ^b^OR, odds ratio; ^c^CI, confidence interval; ^d^ns, not significant; ^e^BMI, body mass index; ^f^PH, personal history; ^g^FH, familiar history.

Logistic regression did not show a good diagnostic throughput because, although the analysis correctly classified 96.9% of the pregnant women, only 37.7% of GDM cases were correctly classified.

**Figure 1:**
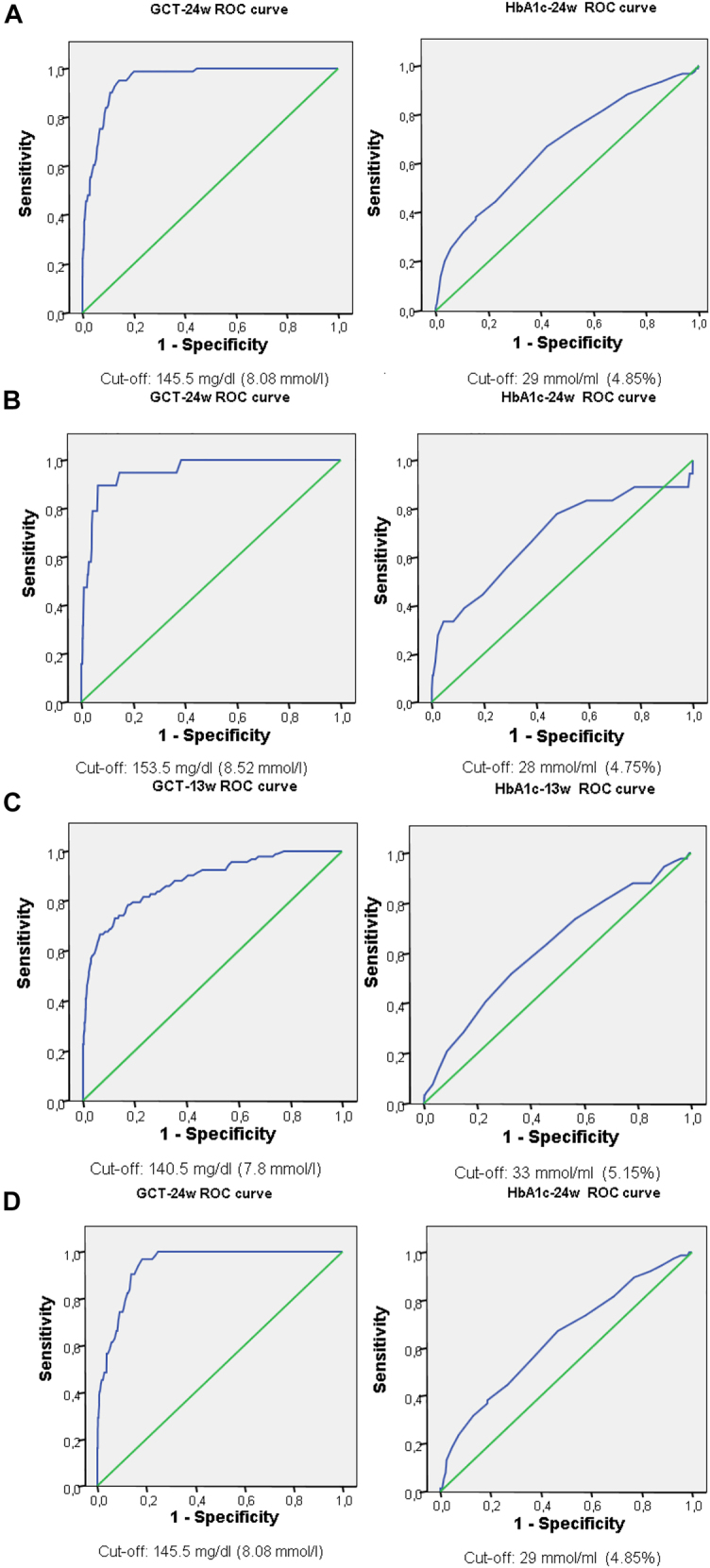
ROC curves showing the sensitivity and specificity of GCT^a^ and HbA_1c_^b^ in detecting GDM^c^. (A) The sensitivity and specificity of GCT-24w^d^ and HbA_1c_-24w^e^ in the whole population. (B) The sensitivity and specificity of GCT-24w and HbA_1c_-24w in the subgroup without risk factors. (C) The sensitivity and specificity of GCT-13w^f^ and HbA_1c_-13w^g^ in the subgroup with risk factors. (D) The sensitivity and specificity of GCT-24w and HbA_1c_-24w in the subgroup with risk factors.

^a^GCT, 50 g glucose challenge test; ^b^HbA_1c_, glycated haemoglobin test; ^c^GDM, gestational diabetes mellitus; ^d^GCT-24w, 50 g glucose challenge test at week 24th; ^e^HbA_1c_-24w, glycated haemoglobin test at week 24th; ^f^GCT-13w, 50 g glucose challenge test at week 13th; ^g^HbA_1c_-13w, glycated haemoglobin test at week 13th.

In terms of diagnostic accuracy, the GCT showed a higher AUC than the HbA_1c_; (0.953 vs. 0.672, respectively) (Figure 1A; Table 4). The best GCT-24w cut-off was 145.5 mg/dL (8.08 mmol/L) (Sensitivity: 95.1%; Specificity: 85.7%; PPV: 22.19%; NPV: 99.75%). For HbA_1c_-24w, the best cut-off was 29 mmol/mol (4.85%) (Sensitivity: 67%; Specificity: 57.8%; PPV: 7.6%; NPV: 97.12%) (Table 4).

**Table 4:** Diagnostic accuracy statistics for each strategy.

Population	Test	Sb^a^	Sp^b^	PPV^c^	NPV^d^	AUC^e^ (95% CI^f^)
All women	GCT-24w^g^≥140 mg/dL (≥7.8 mmol/L)	98.8	79.9	60	99.91	0.953 (0.938–0.968)
	GCT-24w>145.4 mg/dL (>8.07 mmol/L)	95.1	85.7	22.19	99.75	0.953 (0.938–0.968)
	HbA_1c_-24w^h^>29 mmol/mol (>4.84%)	67	57.8	7.6	97.12	0.672 (0.612–0.731)
Women without risk factors	GCT-24w≥140 mg/dL (≥7.8 mmol/L)	94.7	83.7	72	99.88	0.953 (0.915–0.992)
	GCT-24w>153.4 mg/dL (>8.51 mmol/L)	89.5	93.6	20.48	99.79	0.953 (0.915–0.992)
	HbA_1c_-24w>28 mmol/mol (>4.74%)	77.8	52.1	2.87	99.22	0.688 (0.541–0.834)
	Combined algorithm 24w^i^	77.8	95.2	22.95	99.57	
Women with risk factors	GCT-13w≥140 mg/dL (≥7.8 mmol/L)	73.1	87.0	49	96.68	0.882 (0.843–0.921)
	GCT-13w^j^>140.4 mg/dL (>7.79 mmol/L)	73.1	87.7	39.3	96.75	0.882 (0.843–0.921)
	HbA_1c_-13w^k^>33 mmol/mol (>5.14%)	51.6	67.3	14.74	92.65	0.624 (0.562–0.686)
	Combined algorithm 13w^l^	67.8	89.1	40.39	96.21	
	GCT-24w≥140 mg/dL (≥7.8 mmol/L)	100	75.3	60	100	0.944 (0.925–0.962)
	GCT-24w>145.4 mg/dL (>8.07 mmol/L)	96.8	82.7	27.9	99.71	0.944 (0.925–0.962)
	HbA_1c_-24w>29 mmol/mol (>4.84%)	67.1	53.3	11.69	94.62	0.642 (0.575–0.709)
	Combined algorithm 24w	90	83.4	28.42	99.12	

^a^Sb, sensitivity; ^b^Sp, specificity; ^c^PPV, positive predictive value; ^d^NPV, negative predictive value; ^e^AUC, area under the curve; ^f^CI, confidence interval; ^g^GCT-24w, 50 g glucose challenge test at week 24th; ^h^HbA_1c_-24w, glycated haemoglobin at week 24th; ^i^combined algorithm 24w, combined algorithm at week 24th; ^j^GCT-13w, 50 g glucose challenge test at week 13th; ^k^HbA_1c_-13w, glycated haemoglobin test at week 13th; ^l^combined algorithm 13w, combined algorithm at week 13th.

### Analysis of the population without risk factors

A total of 1,054 pregnant women did not have risk factors. The prevalence of GDM in this population was 1.8%. The values of the GCT and HbA_1c_ at week 24 were significantly higher in pregnant women who developed GDM than in those who did not. No difference was found for age (Table 2).

Logistic regression did not show a good diagnostic performance because, even though the analyses correctly classified 98.4% of pregnant women, only 22% of GDM cases were correctly classified.

Concerning diagnostic accuracy, the GCT had a higher AUC than the HbA_1c_ test (0.953 vs. 0.688, respectively) (Figure 1B). The best GCT cut-off was 153.5 mg/dL (8.52 mmol/L) (sensitivity: 89.5%; specificity: 93.6%; PPV: 20.48%; NPV: 99.79%). For HbA_1c_, the best cut-off was 28 mmol/mol (4.75%) (sensitivity: 77.8%; specificity: 52.1%; PPV: 2.87%; NPV: 99.22%) (Table 4). Moreover, two extreme HbA_1c_ thresholds were determined in this population. An HbA_1c_ threshold of 25 mmol/mol (4.45%), showed similar sensitivity to that of the GCT; so, it could be possible to avoid GCT in women with values ​​below this threshold (sensitivity 88.9%). On the other hand, a value of 37 mmol/mol (5.55%) showed a specificity of 98.6% to diagnose GDM.

### Analysis of women presenting risk factors

A total of 953 pregnant women had risk factors. The prevalence of GDM in this population was 10%. The values of the GCT-13w, HbA_1c_-13w, GCT-24w and HbA_1c_-24w were significantly higher in pregnant women who developed GDM than in those who did not. There was no difference concerning age. Similarly, women who developed GDM often presented a BMI>30 kg/m^2^ and had a history of previous GDM. There were no significant differences regarding the variables age >35 years old, macrosomia and family history (Tables 2 and 3).

The logistic regression at both, week 13 and week 24 did not show good diagnostic performance because, although the analyses correctly classified 98.9% of pregnant women, only 52% of GDM cases were correctly classified.

In assessing diagnostic accuracy at week 13, the GCT showed an AUC of 0.882 and the best cut-off was 140.5 mg/dL (7.8 mmol/L) (sensitivity: 73.1%; specificity: 87.7%; PPV: 39.3%; NPV: 96.75%). The HbA_1c_ test showed an AUC of 0.624 and the best cut-off was 33 mmol/mol (5.15%) (sensitivity 51.6%; specificity: 67.3%; PPV: 14.74%; NPV: 92.65%) (Figure 1C, Table 4). About the extreme thresholds, the value 26 mmol/mol (4.55%) was 94.5% sensitive to rule-out GDM and the value 39 mmol/mol (5.75%) was 98.2% specific to diagnose GDM.

When assessing diagnostic accuracy at week 24, the GCT had an AUC of 0.944 and the best cut-off was 145.5 mg/dL (8.08 mmol/L) (sensitivity: 96.8%; specificity: 82.7%; PPV: 27.90%; NPV: 99.71%). The HbA_1c_ test had an AUC of 0.642 and the best cut-off was 29 mmol/mol (4.85%) (sensitivity: 67.1%; specificity: 53.3%; PPV: 11.69%; NPV: 94.62%) (Figure 1D, Table 4). Concerning the extreme thresholds, the value 24 mmol/mol (4.35%) was 94.7% sensitive to discard GDM and the value 39 mmol/mol (5.75%) was 98.7% specific to diagnoses GDM.

### Diagnostic validity of the proposed models with real data

With the data obtained two different strategies were developed for screening GDM: 1) raising the GCT cut-off to reduce the number of women in whom to perform OGTT; 2) using an algorithm that combines a sensitive HbA_1c_ cut-off to rule-out GDM followed by a raised GCT cut-off.

#### Pregnant women without risk factors (n=1,054)

According to the classical approach, the GCT had to be performed to all pregnant women without risk factors, and 187 of these underwent an OGTT. The prevalence of GDM in the population with <140 mg/dl (<7.77 mmol/L) plasma glucose (n=866) was 0.1% (one case).

#### Improved standard procedure (to raise the GCT cut-off to 153.5) (Figure 2A)

**Figure 2:**
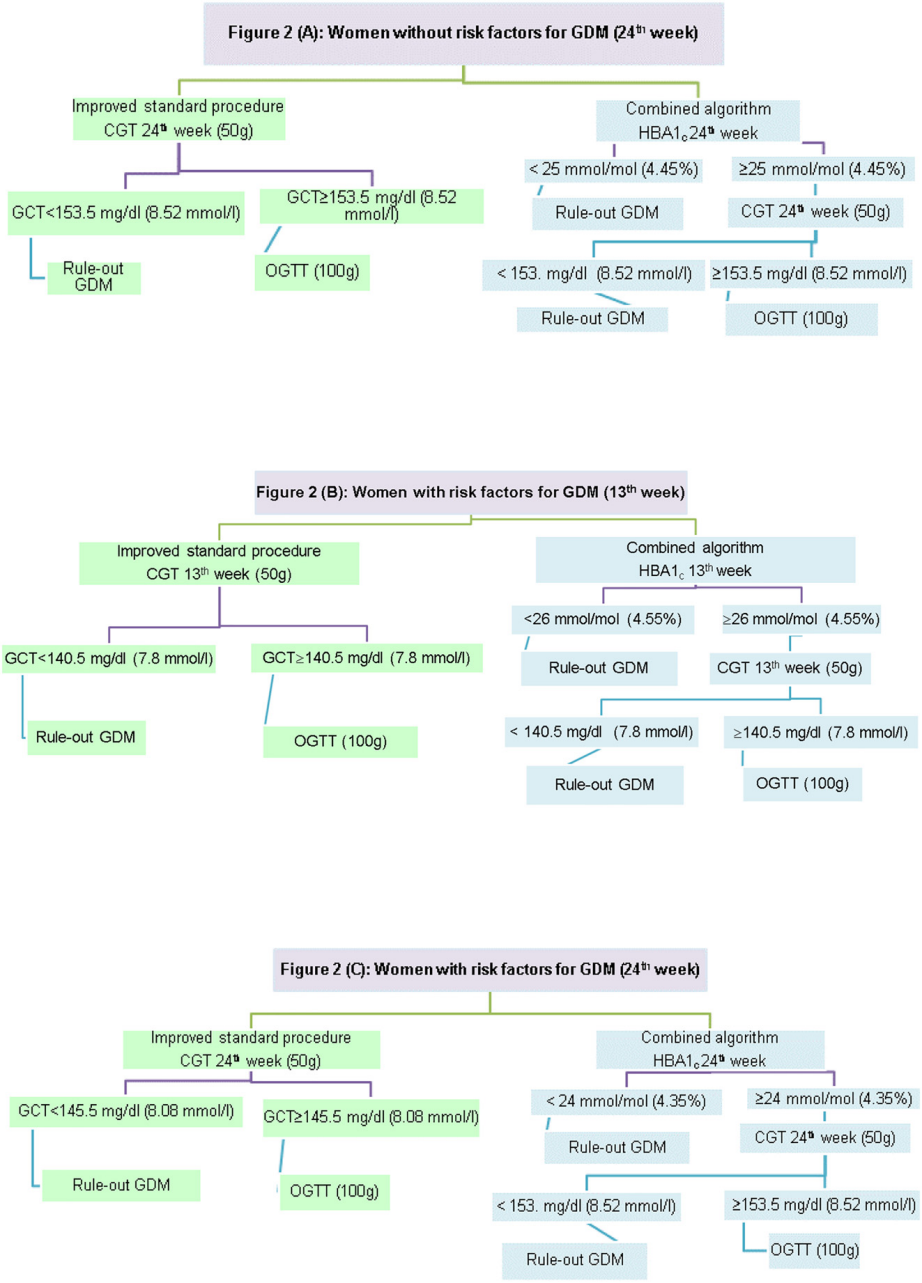
Workflow of the two strategies for the diagnosis of GDM.

GDM, gestational diabetes mellitus; GCT-13th week, 50 g glucose challenge test at week 13th; HbA_1c_-13th week, glycated haemoglobin test at week 13th; GCT-24th week, 50 g glucose challenge test at week 24th; HbA_1c_-24th week, glycated haemoglobin test at week 24th.

With this optimized cut-off, it would only have been necessary to perform the OGTT on 83 women. The prevalence of GDM in the population with <153 mg/dL (<8.49 mmol/L) plasma glucose (n=971) was 0.2% (two cases). The statistical parameters of this model are shown in Table 4.

#### Combined algorithm (Figure 2A)

Theoretically, using an algorithm with the HbA_1c_ threshold of 25 mmol/mol (4.45%) to rule-out GDM, it would have been necessary to perform a 50 g GCT in 782 women. Subsequently, those with a GCT>153.4 (n=61) would have to undergo a 100 g OGTT (49 missing observations from 1,054). In this cohort of pregnant women and considering the final diagnosis, the statistical parameters of the algorithm were: sensitivity: 77.8%, specificity: 95.2%, PPV: 22.95%, and NPV: 99.57% (Table 4).

#### Pregnant women with risk factors (n=953) at week 13

With the classic approach, all women with risk factors followed at week 13 (n=953) required a GCT; after that, using the standard GCT cut-off (140 mg/dL; 7.77 mmol/L), an OGTT had to be performed in 188 pregnant women.

#### Improved standard procedure (to raise the GCT cut-off to 140.5) (Figure 2B)

With this new cut-off, it would have been necessary to perform the OGTT on 182 women. The prevalence of GDM in the population below these threshold values was 3.2%; hence, 25 cases would escape diagnosis. So, raising the GCT cut-off, a negligible benefit was obtained. The statistical parameters of this model are shown in Table 4.

#### Combined algorithm (Figure 2B)

A 26 mmol/mol (4.55%) HbA_1c_ cut-off would allow discarding GDM with 94.5% of sensitivity. Nevertheless, in real conditions of implementation, although it would allow us to rule-out GDM in 89 pregnant women, five cases would scape diagnosis (GDM prevalence of 5.6% in the population with HbA_1c_<26 mmol/mol (4.55%)). Subsequently, those with a GCT>140.4 (n=151) would have to undergo a 100 g OGTT (missing observations 32 from 953). Considering the final diagnosis, the statistical parameters of this algorithm were Sensitivity: 67.8%, Specificity: 89.1%, PPV: 40.39%, NPV: 96.21% (Table 4).

#### Pregnant women with risk factors (n=901) at week 24

With the classic approach, all women with risk factors followed at week 24 (n=901) required a GCT and after that, using the standard GCT cut-off (140 mg/dL; 7.77 mmol/L), 272 women required a 100 g OGTT.

#### Improved standard procedure (to raise the GCT cut-off to 145.5) (Figure 2C)

A total of 215 women had a GCT>145.4; therefore, an OGTT should be performed theoretically. In this population, the prevalence of GDM was 27.9% (60 cases). In the group with GCT<145.5, the prevalence of GDM was 0.3% (only two cases). The statistical parameters of this model are shown in Table 4.

#### Combined algorithm (Figure 2C)

With the combined approach, the 24 mmol/mol (4.35%) HbA_1c_ cut-off would allow us to discard GDM (sensitivity 94.7%). In practice, this cut-off would allow us to rule-out GDM in 100 pregnant women, but four cases would scape diagnosis (GDM prevalence of 4% in the population with HbA_1c_<24 mmol/mol (4.35%)). Subsequently, the 50 g GCT would have to be performed on 801 pregnant women. Of those, 190 showed a GCT≥145.5 and required 100 g OGTT (missing observations 23 from 901). Considering the final diagnosis, the statistical parameters of this algorithm were Sensitivity: 90.0%, Specificity: 83.4%, PPV: 28.42%, NPV: 99.12%.

#### Extreme cut-off approach for early diagnose of GDM in high-risk women

Using a cut-off with a theoretical specificity for diagnosing diabetes of 98.2%, 39 mmol/mol (5.75%) in our population we would diagnose 20 cases, but the actual prevalence of GDM in this subgroup was only 25% (five cases).

## Discussion

This study determines that the standard procedure (50 g oral GCT) improved with higher cut-offs, offers the greatest diagnostic accuracy statistics for GDM. HbA_1c_ might be useful for this purpose, however, this test has lower diagnostic accuracy than the reference standard. So, although the AUC of the HbA_1c_ test was adequate, it was much lower than that of GCT in all the groups studied. This could largely be due to the low sensitivity of the HbA_1c_ test observed across the groups. The diagnostic accuracy of the HbA_1c_ for GDM has been recently explored [21], [22], [23], [24]. The value of AUC in these studies ranges from 0.62 to 0.72. The common idea resulting from those is that the HbA_1c_ cannot replace tests based on glucose overload, although it could be useful as a screening test [23], [ 24] or to identify high-risk pregnant women for the development of GDM [21]. Indeed, the role of HbA_1c_ in the GDM early diagnose of high-risk women has been address by several studies [26], [27], [28]. For this purpose, these works use an approach based on extreme cut-offs that, by maximizing specificity, would enable GDM to be diagnosed in those cases with values higher than the cut-off. Kattini et al. in a systematic review [28], conclude that a cut-off between 5.7 and 6.4% consistently identifies those patients who will develop GDM. In our study, using an extreme cut-off approach, several HbA_1c_ thresholds were found at both the 13th week and 24th week of pregnancy. Theoretically, these thresholds could correctly identify or rule-out GDM maximizing specificity or sensitivity. The best cut-off for early diagnosis of GDM in high-risk patients was 5.75% (39 mmol/L). However, applying the model to our population, only 25% of cases with values above this value developed GDM. These results are almost identical to those shown in the study by Fong et al. [20], in which 27.3% of pregnant women with an HbA_1c_ value above 5.7%, assessed before the 20th week of pregnancy, developed GDM. Furthermore, this finding is consistent with those reported by Punnose et al. [29] and Walker et al. [30], who conclude that the HbA_1c_ test is neither superior to glucose overload nor cost-effective. Therefore, the low AUC of HbA_1c_ in our study and others [26], [ 29], suggests that these cut-offs may not be appropriate.

In line with other authors [31], [32], [33], a combined approach was proposed. However, this approach differs in that it is a combined three-way strategy where: i) pregnant women are classified in the first obstetric visit (week 12) as low-risk or high-risk per well-known risk factors; ii) an HbA_1c_ test is performed at week 13 and/or week 24 as appropriate to rule-out GDM; iii) for those cases in which the GCT must be performed, the test threshold is raised in both low-risk and high-risk populations to avoid unnecessary OGTT. However, the resulting algorithms showed lower diagnostic performance than the improved standard procedure (Table 4).

At the time of writing, the SARS-CoV-2 infection has become the greatest public health challenge for decades. With the object to avoid a potential virus exposition in this pandemic context, health authorities and scientific societies, have proposed new protocols for the GDM diagnosis mainly based on HbA_1c_ and fasting or random plasma glucose [34], [35], [36], [37]. The efficacy of these approaches in detecting GDM and the potential associated pregnancy complications have been thoroughly examined using data from the Hyperglycaemia and Adverse Pregnancy Outcome (HAPO) study [38]. This work convincingly demonstrates that the approaches based on HbA_1c_ and/or fasting plasma glucose [34], [ 35] are associated with an increase of women with GDM not diagnosed, which have significantly higher rates of pregnancy complications. Nevertheless, the approach based on fasting glucose followed by selective OGTT [36], although associated with an increase of women with missed GDM, is not followed by higher rates of adverse outcomes. Thereupon, the authors recommend health authorities and clinicians to balance the risk/benefit of each proposal in the context of the COVID-19 pandemic.

The present study determines that the standard GCT cut-off (140 mg/dL: 7.77 mmol/L) should be reconsidered for all the groups. For women without risk factors, the new GCT value is particularly relevant (153.5 mg/dL: 8.52 mmol/L). Increasing the specificity may be detrimental for the sensitivity of the test. Nevertheless, this higher cut-off point showed sensitivity of 89.5%, and under real application conditions, the missed GDM would rise slightly from 0.1% with the traditional cut-off point to 0.2% with the optimized one. Likewise, adjusting the GCT cut-off to 145 mg/dL (8.05 mmol/L) has been proposed in twin pregnancies to avoid false-positive GDM diagnoses [39], considering low community prevalence rates, as it is the case in our sample group. Additionally, the improved strategy could save time and money. In the low-risk population, we could have saved 55.6% of OGTT. In the high-risk population, raising the GCT cut-off at 13th week showed little benefit, and we could have saved only 3.2% of OGTT; nevertheless, at 24th week the advantage was evident and, 21% of OGTT could have been avoided.

As a crucial element, the present study has been performed with one of the largest sample sizes published to date. On the contrary, the main limitation lies in the different methodological approach to the GDM diagnosis in Spain with respect to those recommended by the ADA or the WHO, which makes it somewhat more difficult to extrapolate directly the findings. As another limitation, it was conducted in a single centre with a 5.7% prevalence of GDM. Multiple pregnancies were not excluded (prevalence<1.5%). Although there was 9.7% of tracking losses as described in detail in “Materials and methods”, it is an acceptable rate of loss during follow-up that does not jeopardize the results.

## Conclusions

This study shows that it is possible to optimize the diagnosis of GDM using new cut-offs for GCT. Additionally, it shows that in terms of diagnostic accuracy, the HbA_1c_ test alone or in a three-way combined algorithm is inferior to the improved GCT. Besides, extreme cut-off point approaches for the early diagnoses of GDM in the high-risk population based on HbA_1c_ test are less efficient than the traditional testing or the improved strategy showed herein.

Notwithstanding, further research on GCT cut-off values are required to gain a better understanding of this issue and make real changes in our GDM diagnostic protocols.

## Supplementary Material

Supplementary Material
