# Supplementary material for: Improved gestational diabetes screening protocol
Source: Adv Lab Med. 2021 Feb 17;2(1):87–96. doi: 10.1515/almed-2020-0072 (PMC10197282; doi:10.1515/almed-2020-0072)
Supplement: Supplementary file 1 — Supplementary Material [file j_almed-2020-0072_suppl.docx]

**Supplemental material:**

**Laboratory procedures.**

For the HbA_1c_ test, venous blood samples were collected in EDTA tubes and measured by high-performance liquid chromatography (HPLC) in a Variant II TURBO Instrument (Bio-rad, Hercules, California). This system is certified by the National Glycohaemoglobin Standarization Program (NGSP) and traceable to “Diabetes Control and Complication Trial” (DCCT).

In order to avoid preanalytical bias related to glycolysis, for glucose testing, venous blood samples were collected in tubes with sodium fluoride as a glycolysis inhibitor [16]. For the GCT, venous blood samples were collected one hour after challenge with 50 g of oral glucose. For the OGTT, venous blood samples were collected at baseline and one, two and three hours after challenge with 100 g of oral glucose. Plasma glucose was measured by the glucose oxidase method in a Cobas 8000 modular analyser (Roche Diagnostic, Basel, Switzerland).

GD was diagnosed according to the criteria of the GEDE [10] and the National Diabetes Data Group (NDDG) [11].

**Statistical analysis**.

The Kolmogorov-Smirnov test was used to assess the normal distribution of data. For the descriptive statistics, the mean, median, standard deviation and interquartile range of the continuous variables (age, HbA_1c_, GCT) and the frequencies and percentages of the discrete variables (each risk factor for GDM) were calculated.

To assess differences between the continuous variables, the Mann-Whitney U test was used. For differences between the discrete variables, Pearson´s chi-square Χ^2^ test was used. Results were expressed in odds ratios (OR), 95% confidence interval (CI) and mean ± standard deviation. Significance was defined as a *p*-value of <0.05.

Finally, a multivariable logistic regression analysis was performed with all the variables.

To determine the diagnostic validity of HbA_1c_ and GCT, sensitivity, specificity, positive predictive value (PPV), negative predictive value (NPV), the receiver operating curve (ROC), and the area under the curve (AUC) were calculated. Moreover, to maximize the specificity (to rule in GDM) and the sensitivity (to rule out GDM), extreme cut point analysis was performed. The statistical analysis was performed on the whole population, as well as on the subgroups with and without risk factors. SPSS v15 statistical software (SPSS Inc., Chicago, Illinois) was used for the statistical analysis.

**Strategies for diagnosing GDM**

With the data obtained, two different strategies were developed for screening GDM in a second stage of the study: strategy 1) raising the screening test cut-off to reduce the number of women in whom to perform OGTT; strategy 2) using an algorithm that combined a sensitive extreme HbA_1c_ breakpoints to rule out diabetes, followed by a raised GCT cut-off. By using these different strategies in our cohort and considering the final diagnosis, it was possible to verify which strategy was the most appropriate. Namely, the one that was at least equal to the conventional strategy but with fewer drawbacks. Additionally, the extreme cut-off approach that maximizes specificity to accurately diagnose GDM was virtually applied to the group of women at high-risk in the first trimester of pregnancy.
